# An Integrative Transcriptomic and Metabolomic Analysis of Red Pitaya (*Hylocereus polyrhizus*) Seedlings in Response to Heat Stress

**DOI:** 10.3390/genes12111714

**Published:** 2021-10-27

**Authors:** Zhengli Jiao, Weijuan Xu, Quandong Nong, Mei Zhang, Shuguang Jian, Hongfang Lu, Jiantong Chen, Mingyong Zhang, Kuaifei Xia

**Affiliations:** 1 Guangdong Provincial Key Laboratory of Applied Botany & Key Laboratory of South China Agricultural Plant Molecular Analysis and Genetic Improvement, South China Botanical Garden, Chinese Academy of Sciences, Guangzhou 510650, China; jiaozl@scbg.ac.cn (Z.J.); xuweijuan@scbg.ac.cn (W.X.); nongquand@163.com (Q.N.); zhangmei@scbg.ac.cn (M.Z.); zhangmy@scbg.ac.cn (M.Z.); 2 University of Chinese Academy of Sciences, Beijing 100049, China; 3 Wenshan Academy of Agricultural Sciences, Wenshan 663000, China; 4 CAS Engineering Laboratory for Vegetation Ecosystem Restoration on Islands and Coastal Zones, South China Botanical Garden, Chinese Academy of Sciences, Guangzhou 510650, China; jiansg@scbg.ac.cn (S.J.); luhf@scbg.ac.cn (H.L.); cjt0528@scbg.ac.cn (J.C.); 5 Research Station of Vegetation Ecosystems on Coral Islands in South China Sea, Guangzhou 510650, China

**Keywords:** red pitaya, heat stress, transcriptome, metabolome, PR-1 protein

## Abstract

Red pitaya (*Hylocereus polyrhizus*) is a significant functional food that is largely planted in Southeast Asia. Heat stress (HS) induced by high temperatures is likely to restrict the growth and survival of red pitaya. Although pitaya can tolerate temperatures as high as 40 °C, little is known of how it can withstand HS. In this study, the transcriptomic and metabolomic responses of red pitaya seedlings to HS were analyzed. A total of 198 transcripts (122 upregulated and 76 downregulated) were significantly differentially expressed after 24 h and 72 h of exposure to 42 °C compared with a control grown at 28 °C. We also identified 64 differentially accumulated metabolites in pitaya under HS (37 increased and 27 decreased). These differential metabolites, especially amino acids, organic acids, and sugars, are involved in metabolic pathways and the biosynthesis of amino acids. Interaction network analysis of the heat-responsive genes and metabolites suggested that similar pathways and complex response mechanisms are involved in the response of pitaya to HS. Overexpression of one of the upregulated genes (*contig10820*) in *Arabidopsis*, which is a homolog of *PR-1* and named *HuPR-1*, significantly increased tolerance to HS. This is the first study showing that *HuPR-1* plays a role in the response of pitaya to abiotic stress. These findings provide valuable insights that will aid future studies examining adaptation to HS in pitaya.

## 1. Introduction

Global ambient temperature has gradually increased because of greenhouse gases such as CO_2_, methane, chlorofluorocarbons, and nitrous oxides. Heat stress (HS) is usually defined as a temperature increase above a threshold level for a period of time sufficient to cause irreversible damage to plant growth and development. It occurs in response to a transient increase in temperature, usually 10–15 °C above environmental temperature. HS associated with increases in ambient temperature globally poses a serious threat to the growth and production of plants. Plants have evolved various physiological and biochemical adaptations to avoid or reduce the damage caused by HS [1]. HS is one of the most crucial forms of abiotic stress, as it disrupts homeostasis, limits plant growth and development, and even leads to death [2,3]. Elucidating the mechanisms by which plants respond to HS is thus critically important. 

HS has independent effects on the physiology and metabolism of plant tissues and cells. Many types of physiological damage are observed under heat stress, such as the scorching of leaves and stems, leaf abscission and senescence, shoot and root growth inhibition, and fruit damage, all of which decrease plant productivity [4]. Higher plants undergo a series of cellular and metabolic reactions to withstand high temperatures, which include changes in the cellular structure and organization, such as organelles and cytoskeleton, as well as membrane functions [5]; reductions in normal protein synthesis; increases in the transcription and translation of heat shock proteins (HSPs) and heat shock transcription factors (HSFs) [6]; and production of phytohormones such as abscisic acid (ABA), antioxidant substances, and other protective molecules [7]. Some secondary metabolites are also involved in the responses of plants to HS, such as phenolics (e.g., flavonoids, anthocyanins, and plant steroids) [1]. HS activates MAPKs, which regulate HSP gene expression; MAPK activation may be related to heat-induced changes in membrane fluidity and calcium signaling, which are significant for HSP gene expression and heat tolerance [8]. HS causes changes in respiration and photosynthesis, leading to shortened life cycles and reduced plant productivity [9]. High temperature alters the activities of carbon metabolism enzymes, starch accumulation, and sucrose synthesis by downregulating specific genes that participate in carbohydrate metabolism [10]. 

Pitaya (Cactaceae: *Hylocereus*) is a nutritious tropical fruit with high commercial and medical value [11,12]. It has recently begun to be cultivated in Thailand, the Philippines, Vietnam, Malaysia, and China. Pitaya can be cultivated in diverse climates because of its tolerance of various types of environmental stress, such as drought, heat, salt, and poor soil [13]. Most research on pitaya in previous decades has mostly concentrated in the betaine synthesis process, mainly including their purification and identification [14,15], their physical and chemical properties [16], and their antioxidant and radical-scavenging capacity [17]. Metabolite profiling of pitayas has led to the identification of several betalain biosynthesis-related compounds [18]. In addition, transcriptomic analysis has led to the identification of several key genes in the betalain biosynthesis pathway [19]. Recently, transcriptome and proteomics levels have been used to investigate the molecular mechanism of pitaya response to different types of abiotic stress such as salt, drought, and cold stress [20,21,22]. Proteomic analysis of pitaya led to the identification of 116 differentially abundant proteins, which were mainly related to chloroplast and mitochondria metabolism, indicating that they play a critical role in coping with cold stress [22]. Until now, only a few stress-associated genes, including miR396b-GRF, *HuCAT3*, and *HuERF1* in pitaya, have been proved to enhance tolerance to cold, drought, and salt stress [23,24,25]. However, genomic resources and genetic information of pitaya are still scarce. More genetic data are needed to aid studies examining the resistance of pitaya to abiotic and biotic stress and crop breeding.

The physiological and molecular mechanisms underlying the response of plants to HS are complex and depend on diverse signal transduction pathways, genes, and metabolites [26,27]. Transcriptomic analysis is an effective and widely used technique to identify genes associated with heat tolerance [28,29,30]. Many heat-responsive genes have been identified, including HSPs, HSFs, WRKYs, MYBs, and NACs [31,32,33]. Metabolomic analysis has become an effective means to study plant responses to biotic and abiotic stress [34,35,36]. An integrative transcriptomic and metabolomic analysis of pitaya in response to HS could provide novel insights into the mechanism by which plants respond to HS.

**Figure 1:**
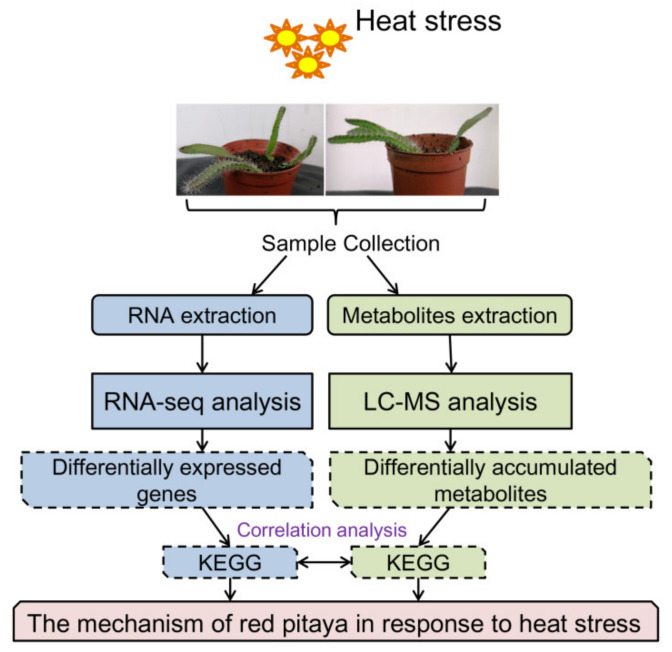
Flow chart of the experimental process. Blue represents the transcriptome process, and green represents the metabolome process.

The optimum temperature for the growth of pitaya is 20 to 30 °C, and it can tolerate temperatures as high as 40 °C. However, little is known about how pitaya can withstand such high temperatures. Here, we analyzed the transcriptomic and metabolomic responses of pitaya seedlings after 24 h and 72 h of exposure to 42 °C and 28 °C to identify heat tolerance genes and metabolites and characterize the mechanisms underlying the response of pitaya to HS (Figure 1). Our study suggests that many heat-responsive genes and metabolites are participating in the response to HS and that overexpression of up-regulated genes (*HuPR-1*) can improve the heat tolerance of transgenic *Arabidopsis* plants. 

## 2. Materials and Methods

### 2.1. Plant Materials and Heat Treatments

Red pitaya (*Hylocereus polyrhizus*) were purchased online. After surface-sterilization, seeds of pitaya were sown in the soil in plastic pots and grown to the three-month-old seedling stage in a greenhouse under controlled conditions (14 h/10 h day/night cycle, 28 ± 1 °C, and 60% ± 5% relative humidity). For heat treatments, plants were subjected to 42 °C for 24 h and 72 h, and control plants were cultivated under normal conditions. Both heat stress and control treatments consisted of three independent biological replicates. A total of 9 samples were harvested in liquid nitrogen and stored at −80 °C for RNA extraction.

### 2.2. RNA Isolation and Library Preparation for Transcriptomic Analysis

Total RNA from different samples was extracted using TRIzol reagent (Invitrogen, Waltham, MA, USA) according to the manufacturer’s instructions. RNA quality and quantity, including the RNA integrity number (RIN), were analyzed using 1% agarose gel electrophoresis and an RNA Nano 6000 Assay Kit with an Agilent Bioanalyzer 2100 system (Agilent Technologies, Santa Clara, CA, USA), respectively [37]. mRNA was purified with oligo (dT) beads; these cleaved RNA fragments were then used as templates to synthesize first-strand cDNA using random hexamer primers, followed by second-strand cDNA synthesis using RNaseH and DNA polymerase I. Illumina Hiseq platform was used for paired-end cluster generation and sequencing [37].

### 2.3. Sequencing, De Novo Assembly, and Annotation

RNA sequencing libraries were generated using the NEBNext® Ultra™ RNA Library Prep Kit for Illumina® (New England Biolabs, Ipswich, MA, USA) according to the manufacturer’s instructions. The produced libraries were sequenced using Illumina HiSeq^TM^ 2500 at Biomarker Technologies Co. Ltd. (Beijing, China). Raw sequencing data in FASTQ format were processed by *NGSToolkits* (version.2.3.3) using default parameters [38]. Clean high-quality data were obtained by removing reads containing adapters, poly-Ns, or low-quality reads from the raw data. The Trinity method was used for the de novo assembly of high-quality data to generate unigenes [39]. 

The unigenes were aligned to a series of databases (the NCBI non-redundant (Nr) protein database (NR, Jan, 2013), Swiss-Prot protein database (Swissprot, The European, Bioinformatics Institute, Cambridge, UK), Cluster of Orthologous Genes database (NCBI, Bethesda, MD, USA), KEGG pathway database (Kanehisa Laboratories, Kyoto, Japan), and the GO database) using BLASTx to obtain annotation and classification information. 

### 2.4. Identification of DEGs

DEGs (differentially expressed genes) were identified by following previously described methods [37]. The TopHat program (http://tophat.cbcb.umd.edu/, accessed on 13 February 2021) was used to map the clean data to the de novo assembled reference transcriptome. DEGs of different libraries were analyzed using edgeR software [40]. Data were considered high quality on the basis of the following criteria: log_2_ FC (fold change) ≥ 1, the count value for each gene in the two datasets > 20, and the adjusted *p*-value FDR (false discovery rate) ≤ 0.001.

### 2.5. Transcriptome Assembly and DEG Validation

To validate the accuracy of the assembly, we amplified the CDS regions of nine randomly selected transcripts from the de novo transcriptome by PCR. PCR reactions procedures followed those of Nong et al. (2019). qRT-PCR amplification was conducted using a Roche LightCycler 480 Gene Scanning system (Roche, Basel, Basel-Stadt, Switzerland) to validate the accuracy of the RNA-seq data following previously described methods [41]. The expression levels of selected DEGs were normalized by comparison with the internal reference gene *UBQ* [42]. The relative expression levels of each transcript were calculated using the 2^−ΔΔCt^ method [43]. There were three biological and three technical replicates per treatment. The primers used for PCR and qRT-PCR were designed using Primer Premier 5 (Premier Biosoft, San Francisco, CA, USA). All primers are listed in Table S1.

### 2.6. Extraction of Metabolites

Metabolites were extracted and detected by Biomarker Technologies (Beijing, China). Control and 24 h heat-treated seedlings of red pitaya were collected, ground to a uniform powder using liquid nitrogen, and stored at −80 °C. Samples were thawed at 4 °C on ice, and 100 mg was placed in a 1.5 mL centrifuge tube. After extraction with 300 μL methanol, 20 μL of internal standard substances was added, followed by vortexing for 30 s. The mixtures were then treated with ultrasound for 10 min (incubation with ice water) and incubated for 1 h at −20 °C. The supernatant was transferred to a new 1.5 mL centrifuge tube. After centrifugation at 13,000 rpm for 15 min (4 °C), 200 μL of the supernatant was transferred to a conical insert of a 2 mL LC–MS glass vial. A total of 20 μL of the supernatant from each sample was mixed as a pooled QC sample. Finally, 200 μL of the supernatant was used for the UHPLC–QTOF–MS analysis.

### 2.7. LC–MS/MS Analysis

LC–MS/MS analysis was performed on an UHPLC system (1290, Agilent Technologies, Santa Clara, CA, USA) with a UPLC BEH Amide column (1.7 μm, 2.1 × 100 mm, WatersMilford, MA, USA) combined with a TripleTOF 5600 system (Q-TOF, AB Sciex, Framingham, MA, USA). The mobile phase consisted of 25 mM NH_4_OAc and 25 mM NH_4_OH in water (pH = 9.75) (A) and acetonitrile (B), and the elution gradient was as follows: 0 min, 95% B; 7 min, 65% B; 9 min, 40% B; 9.1 min, 95% B; 12 min, 95% B. The flow rate was 0.5 mL/min, and the injection volume was 3 μL. The Triple TOF mass spectrometer was used to obtain MS/MS spectra through information-dependent acquisition during LC–MS experiments. In this mode, the acquisition software (Analyst TF 1.7, AB Sciex, Framingham, MA, USA) can continuously evaluate the full scan survey MS data, as the MS/MS spectra are obtained on the basis of preselected criteria. In each cycle, 12 precursor ions with intensities greater than 100 were fragmented with a collision energy (CE) of 30 V (15 MS/MS events with a product ion accumulation time of 50 millisecond each). ESI source conditions were set as follows: ion source gas 1, 60 Psi; ion source gas 2, 60 Psi; curtain gas, 35 Psi; source temperature, 650 °C; and ion spray voltage floating, 5000 V or −4000 V in positive or negative modes, respectively.

### 2.8. Data Preprocessing and OPLS-DA

We used ProteoWizard to convert MS raw data files into the mzXML format and processed them by R package XCMS (version 3.2). The preprocessing results generated a data matrix consisting of the retention time (RT), mass-to-charge ratio (*m*/*z*) values, and peak intensity. After XCMS data processing, R package CAMERA was used for peak annotation, and metabolites were identified using the in-house MS2 database.

OPLS-DA was used to analyze the data. The prediction parameters of the evaluation model were R2X, R2Y, and Q2, where R2X and R2Y represent the interpretation rate of the model and the X and Y matrixes, respectively, and Q2 represents the prediction ability of the model. The model is more stable the closer the three indicators are to 1. The model can be considered valid if Q2 > 0.5 and excellent if Q2 > 0.9. For samples with biological replicates, the *p*-value of Student’s *t*-test and the VIP value of the OPLS-DA model were combined to screen the differential metabolites. The criteria were *p* < 0.05 and VIP > 1.

### 2.9. DNA Constructs and Plant Transformation

To obtain a recombinant vector for the overexpression assay in transgenic *Arabidopsis*, we PCR amplified the full-length cDNA of *HuPR-1* using the primer pair HuPR-1-F and HuPR-1-R (Table S1) by PCR. Then, the PCR product was cloned into the *Bgl*II and *Spe*I sites of the pCAMBIA1302-v plasmid (modified from pCAMBIA1302) by homologous recombination, with an expression cassette controlled by the CaMV 35S promoter. The construct was sequenced correctly and transferred into *Agrobacterium tumefaciens* strain GV3101, and the positive clone was selected and cultured. The T-DNA region containing the *HuPR-1* and *NPTII* expression cassette was transformed into *Arabidopsis* using the floral dip method. Seeds of the T_1_ and T_2_ generations were germinated on MS agar medium containing 50 mg/L kanamycin to obtain homozygous lines [25]. Positive transgenic plants were selected according to the segregation ratio (sensitive: resistant = 1:3) and were confirmed by genomic PCR with the primer pair 1302-F/1302-R. The expression levels of *HuPR-1* were detected using qRT-PCR analysis as described above [25].

### 2.10. HS Tolerance Assays in Transgenic Arabidopsis

WT and transgenic seeds were germinated simultaneously on MS medium plates. Plants of each genotype were planted in a greenhouse as mentioned above. For the survival assay, 7-day-old seedlings were heat-treated at 44 °C for 2 h, then returned to 22 °C to grow for 2 days, then photographed, and survival rates were calculated. More than 40 plants of each line were analyzed.

### 2.11. Statistical Analysis

All the experiments in this study were repeated three times, and the data were expressed as mean ± SD. Differences between each transgenic line and WT plants were assessed by Student’s *t*-test in Excel (Microsoft Office 2010). Asterisks indicate significant differences (* *p* < 0.05, ** *p* < 0.01).

## 3. Results

### 3.1. Sequencing and De Novo Assembly of the Pitaya Transcriptome

Nine transcriptome libraries were constructed using Poly-A+ RNA isolated from three-month-old red pitaya seedlings showing normal growth under control conditions (28 °C) or heat treatment (42 °C). These transcriptome libraries were sequenced using the Illumina HiSeqTM 2500 platform, and 78,871,914 paired-end raw reads were generated. The high-quality reads were assembled into 73,589 transcripts with an average length of 2141 bp and an N50 value of 2848 bp by Trinity software. The total sequence length was 96.3 Mb, and the transcript length range was 206–40,937 bp (Table 1 and Table S2).

**Figure 2:**
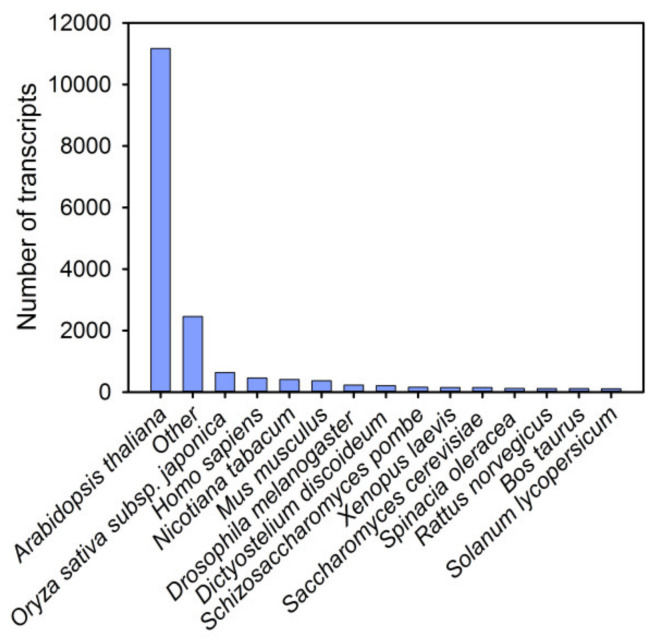
Species distribution of the top BLAST hits for total homologous sequences. Among them, the species with the most hits was *Arabidopsis thaliana*.

In total, 16,856 unique transcripts were successfully mapped to 6215 protein sequences in the Swiss-Prot database, which was associated with 427 species. The species with the most hits was *Arabidopsis thaliana* (66.2%), followed by *Oryza sativa* subsp. *japonica* (3.8%), *Homo sapiens* (2.7%), and *Nicotiana tabacum* (2.4%) (Figure 2 and Table S3). This result indicates that red pitaya is closely related to *Arabidopsis thaliana*, as expected. Similar results were obtained after performing a query against the NCBI RefSeq RNA database, and 17,194 transcripts were successfully mapped (Table S4). 

Nine transcripts were randomly selected for sequencing to confirm the sequence assembly. The coding sequence (CDS) length ranged from 594 bp to 1572 bp. The amplified CDS regions showed a 99–100% identity with their associated pitaya transcripts (Table S5). 

### 3.2. Identification of Differentially Expressed Genes (DEGs) under HS

**Figure 3:**
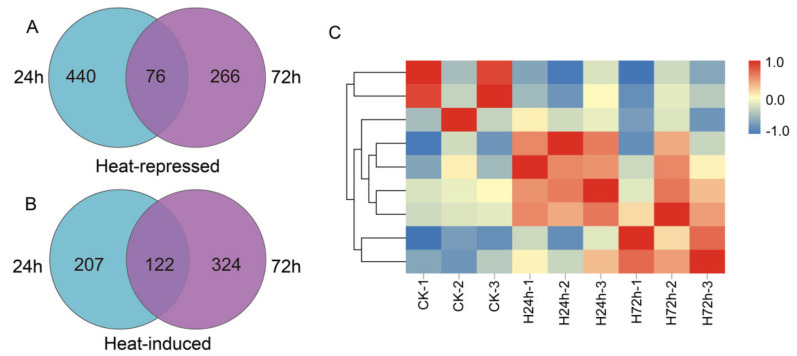
Overview of transcriptome analysis. (**A**) Venn graph for 24 h and 72 h based on heat downregulated (repressed) genes. (**B**) Venn graph for 24 h and 72 h based on upregulated (induced) genes. (**C**) The heatmap of the DEGs in RNA-seq analysis. CK: control for 0 h; H24h: heat stress for 1 h; H72h: heat stress for 72 h.

We identified 845 DEGs between the control and heat-treated samples under the 24 h treatment, and 788 DEGs between the control and heat-treated samples under the 72 h treatment. The expression of 329 and 446 genes was upregulated, and that of 516 and 342 genes was downregulated after 24 h and 72 h of high temperature stress, respectively (Figure 3A,B). Following heat treatment, the expression of 122 transcripts was induced at 24 h and 72 h, and the expression of 76 transcripts was suppressed (Figure 3A,B). To obtain an integral transcriptional profile of the different expressed transcripts under different phases of heat stress, we performed the hierarchical clustering analysis and found that heat stress affected the transcriptional profiles and the number of downregulated/upregulated genes (Figure 3C). Photosynthesis is one of the physiological processes most sensitive to heat. Under HS, the photochemical reaction in the thylakoid sheet and the carbon metabolism in the chloroplast matrix are vulnerable to damage [44,45]. HS disrupts the thylakoid membrane, thereby inhibiting the activities of the membrane-associated electron carriers and enzymes and reducing the rate of photosynthesis [46]. In our study, several DEGs related to the electron carriers and enzymes in the photosynthesis pathway were identified (Table 2). 

Transcription factors (TFs) play a significant role in plants response to HS by regulating the expression of target genes. The major TFs identified from the DEGs involved in the response to HS in this study included 15 HSPs, 8 MYBs, 5 AP2/ERFs, 3 HSFs, 2 bZIPs, and 1 MBF1C gene (Table S6); the expression of seven MYB TFs was suppressed (Table 3), and the expression of 13 HSPs was induced (Table 4).

To verify the RNA-Seq data, we randomly chose seven contigs (genes) from the dataset and verified them by qRT-PCR. The qRT-PCR results showed that the patterns of expression of the selected contigs were consistent with the RNA-Seq dataset (Figure S1). These findings confirm the reliability of the obtained data.

To investigate the mechanisms underlying the expression of DEGs in response to HS, we analyzed their functions through Gene Ontology (GO) analysis. A total of 1434 DEGs were mapped to 888 protein sequences in Swiss-Prot, of which 546 DEGs were uncharacterized transcripts (Table S7). These DEGs were assigned to three classes (molecular function, biological process, and cell component) of GO categories (Table S7 and Figure S2). Among the classified GO groups, the terms such as catalytic activity (GO:0003824), metabolic process (GO:0008152), and cell part (GO:0044464) were dominant in each of the three main categories (Figure S2). We also observed a high level of enrichment of genes in the following functional groups: binding (GO:0005488), cellular process (GO:0009987), response to stimulus (GO:0050896), and organelle (GO:0043226) (Figure S2).

**Figure 4:**
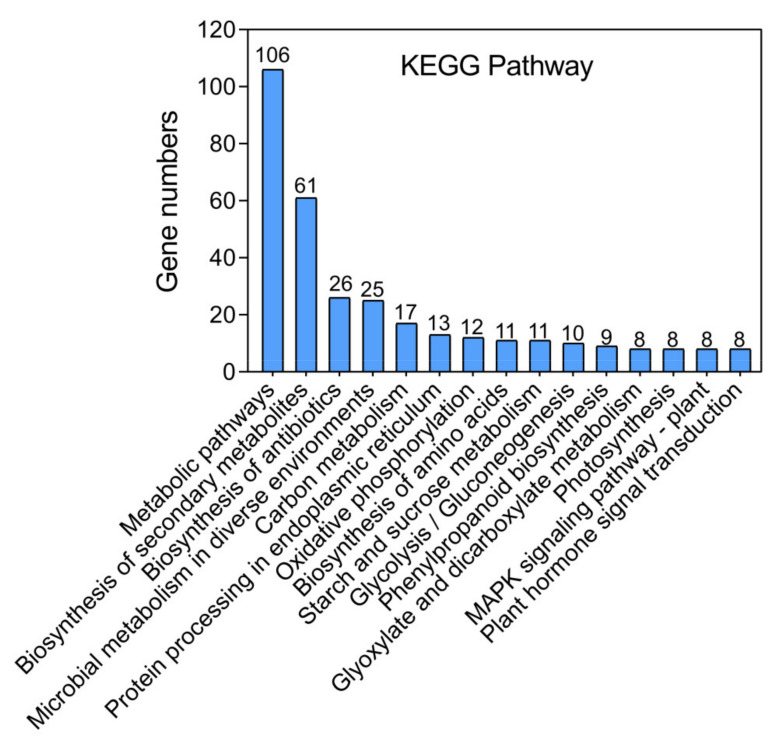
Top 15 of the pathway assignments of red pitaya genes according to the Kyoto Encyclopedia of Genes and Genomes (KEGG) database. DEGs in the heat stress were significantly enriched in the metabolic pathways, biosynthesis of secondary metabolites, biosynthesis of antibiotics, etc.

The Kyoto Encyclopedia of Genes and Genomes (KEGG) pathway analysis provided insight into the complex biological functions of these genes under HS. DEGs in the heat treatment were significantly enriched in the following pathways: “metabolic pathways,” “biosynthesis of secondary metabolites,” “carbon metabolism,” “biosynthesis of antibiotics,” and “protein processing in endoplasmic reticulum” (Figure 4, Table S8). These DEGs were also enriched in “photosynthesis,” “MAPK signaling pathway,” and “plant hormone signal transduction” (Figure 4, Table S8). The above results showed that red pitaya undergoes complex metabolic and enzymatic reactions under HS.

### 3.3. Analysis of the Metabolites in Red Pitaya Seedlings under HS

**Figure 5:**
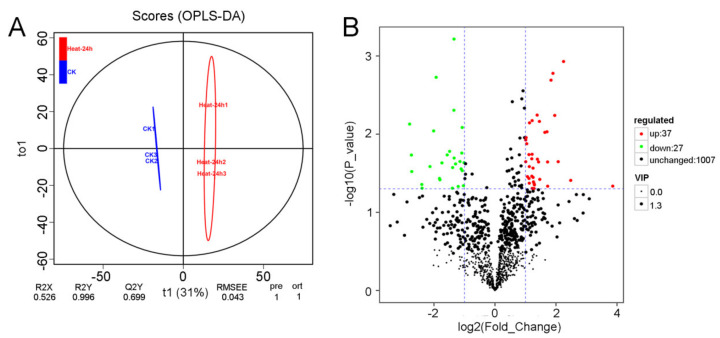
Identification of differentially accumulated metabolites. (**A**) Metabolic analysis using an OPLS-DA model. (**B**) Volcano plots of differentially accumulated metabolites under HS compared with the control group. The green indicates downregulated metabolites, red indicates upregulated metabolites, and black indicates no significant change.

**Figure 6:**
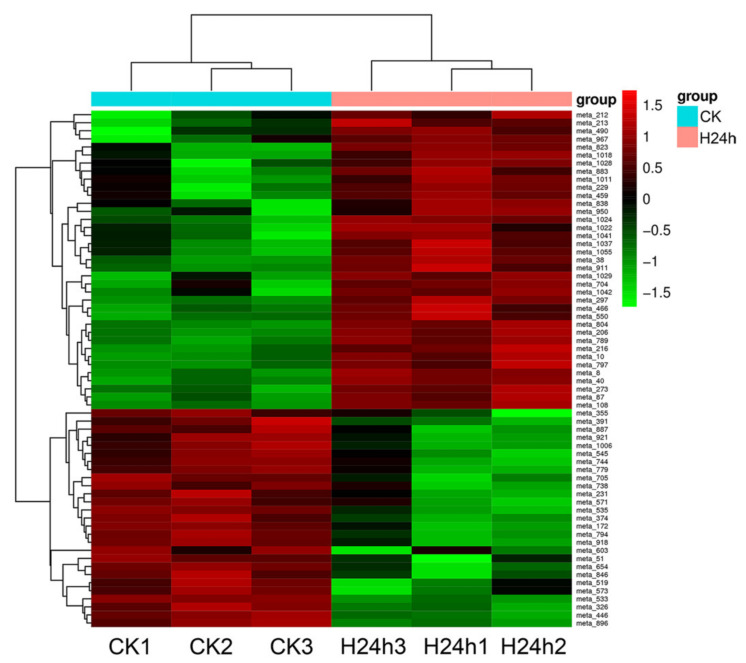
Heatmap of all differentially accumulated metabolites for H24h. CK, the control group; H24h, the 24 h heat stress group.

Metabolic pathway analysis can provide insights into the main biochemical and signal transduction pathways involved in the response of red pitaya to HS. To identify the metabolites in response to heat treatment, we compared the metabolic profiles between normal pitaya seedlings and seedlings subjected to heat treatment for 24 h using LC–MS/MS. We detected a total of 1071 metabolic peaks, with 450 non-target metabolites and 621 target metabolites (Table S9). In the orthogonal projections to latent structures discriminant analysis (OPLS-DA) model, the score (T1) of the main component in the OSC process was 31% (Figure 5A). Compared with the control group (CK), 64 metabolites (37 upregulated and 27 downregulated) were identified to be differentially accumulated under HS (Figure 5B, Table S9). We also generated a heatmap of all differentially accumulated metabolites under HS to display changes in metabolites compared with CK group (Figure 6). Metabolites that increased in content mainly included meta_206, meta_40, meta_10, and meta_8, which represented glycerol tributanoate, cis-aconitate, L-isoleucine, and mesaconic acid, respectively. However, some metabolites were unmapped, including meta_1028, meta_1029, and meta_1037 (Table S9).

**Figure 7:**
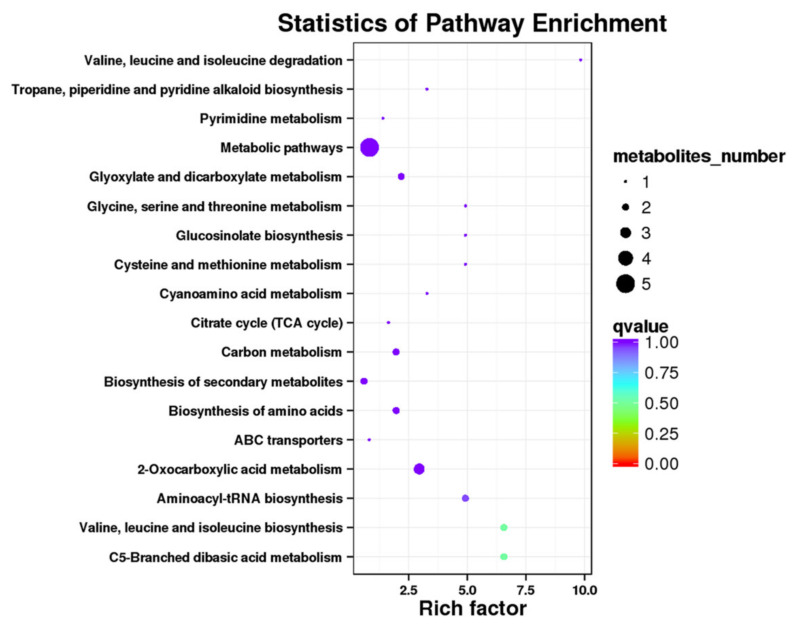
Top 18 enriched pathways for heat-responsive compounds.

To obtain a better understanding of the characteristics of the compounds involved in metabolic processes, we conducted a KEGG enrichment analysis for the detected metabolites. The differentially accumulated metabolites were mapped to different pathways (Table 5 and Table S9). Among these pathways, “metabolic pathways (ko01100),” “biosynthesis of secondary metabolites (ko01110),” “2-oxocarboxylic acid metabolism (ko01210),” “carbon metabolism (ko01200),” and “biosynthesis of amino acids (ko01230)” were the top five enriched pathways (Figure 7). 

### 3.4. Interaction Network Analysis between Heat-Regulated Genes and Metabolites 

Gene–metabolite interaction networks can provide insight into the functional relationships between genes and metabolites and aid in the identification of new regulatory elements [47]. The KEGG analysis showed that the top three pathways were metabolic pathways (ko01100), biosynthesis of secondary metabolites (ko01110), and carbon metabolism (ko01200) (Table S9). Metabonomic analysis revealed that the known differential compounds mainly included meta_40 (cis-aconitate), meta_10 (L-Isoleucine), meta_8 (mesaconic acid), meta_51 (3-phosphoserine), and meta_212 (uridine). Metabolites are the final products of cell activities that directly reflect the effects of environmental changes or physiological and pathological changes on plants. In this study, meta_40 (cis-aconitate) accumulated under HS, which is localized in mitochondria and is an intermediate product of the isomerization of citric acid to isocitrate (ko00020 and ko00630) by aconitase hydratase. The KEGG analysis showed that *contig582* (ACOC) encoded aconitate hydratase (ACO; aconitate hydratase (EC: 4.2.1.3)) in the citrate cycle (TCA cycle) and was upregulated under HS (Table S6). Therefore, the expression of the aconitic acid hydratase gene *contig582* was upregulated under HS; this gene promotes the conversion of citric acid to isocitric acid and thus increases the accumulation of the intermediate product cis-aconitic acid. Meta 40 also participates in other important pathways, including the biosynthesis of secondary metabolites (ko01110), metabolic pathways (ko01100), 2-oxocarboxylic acid metabolism (ko01210), C5-branched dibasic acid metabolism (ko00660), and glyoxylate and dicarboxylate metabolism (ko00630). These findings indicated that cis-aconitate may play an important role in response of red pitaya to HS through its effects on different pathways. The content of other metabolites was also altered under HS, such as meta_51 (3-phosphoserine), which participates in glycine, serine, and threonine metabolism (ko00260). The content of 3-phosphoserine decreased under HS, and affected the expression of the downstream gene *contig28686*, which encodes trpB (tryptophan synthase β chain, (EC: 4.2.1.20)) and catalyzes the transformation of serine to L-tryptophane. We also found several other genes upregulated in this signaling pathway, but their roles in the response to HS remain unclear. The expression of *contig11990* (*ODBA1*, 2-oxoisovalerate dehydrogenase subunit α1) was upregulated under HS in valine, leucine, and isoleucine degradation (ko00280) (Table S6), and the content of meta_10 (L-isoleucine) increased according to the metabolomic analysis; thus, HS might lead to an increase in the content of L-isoleucine and induce the expression of the downstream gene *contig11990*.

### 3.5. Overexpression of HuPR-1 in Arabidopsis Increased Heat Tolerance 

**Figure 8:**
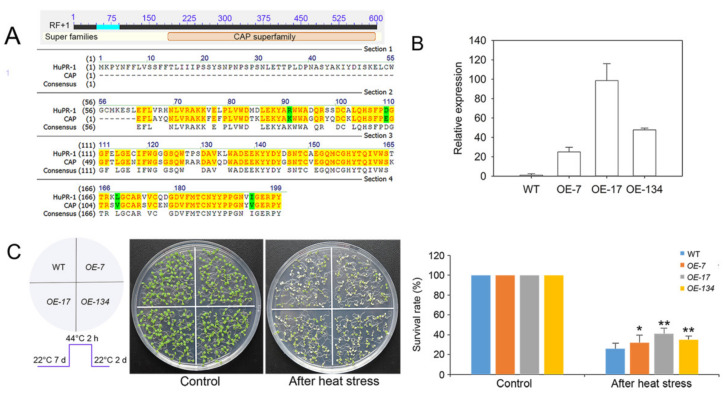
Sequence analysis of *HuPR-1* and its overexpression conferred heat tolerance to transgenic *Arabidopsis* plants. (**A**) Protein sequence alignment result of HuPR-1 in NCBI and protein alignment by Vector NTI Advance 11. (**B**) The qRT-PCR was used to measure the expression levels of *HuPR-1* in transgenic *Arabidopsis* plants. (**C**) Survival rates (%) of WT and transgenic seedlings after heat treatment. Seven-day-old seedlings were heat-treated at 44 °C for 2 h and returned to 22 °C to grow for 2 days, and then photographed; following this, the survival rates were calculated. More than 50 plants of each line were analyzed. Bars represent standard deviations. Asterisks indicate statistically significant differences compared with wild type by Student’s *t*-test (* *p* < 0.05, ** *p* < 0.01).

To verify the genes upregulated in the response of red pitaya to HS, we selected the candidate gene *contig10820* for functional verification; this gene is also upregulated in response to salt stress according to the salt transcriptome database [37]. The expression of *contig10820* was increased by 3.47 times when induced by high temperature and increased by 3.01 times when induced by salt. The CDS of *contig10820* is 594 bp and is a homolog of PR-1 (pathogenesis-related protein 1) family genes. On the basis of the Pfam database, we found that HuPR-1 contained the CAP superfamily (cysteine-rich secretory proteins, antigen 5, and pathogenesis-related 1 proteins (CAP)) domain structure (PF00188) (Figure 8A). To determine its biological function, we generated transgenic *Arabidopsis* plants overexpressing *HuPR-1* that were driven by the CaMV 35S promoter. Three homozygous T_3_ lines (*OE-4*, *OE-17*, and *OE-134*) were selected for *HuPR-1*, and qRT-PCR was performed. *HuPR-1* was highly expressed in all transgenic *Arabidopsis* lines (Figure 8B), and they were used for phenotypic analyses (Figure 8C). For the survival assay under HS, 7-day-old seedlings of wild type (WT) and transgenic lines were heat-treated at 44 °C for 2 h, and then recovered under normal conditions (22 °C) for 2 days (Figure 8C). The survival rates of all transgenic lines were significantly higher than those of WT plants (Figure 8C). These data indicated that *HuPR-1* may play a critical role in the response of pitaya to HS.

## 4. Discussion

Temperature is one of the most significant factors limiting plant growth and development. When plants are subjected to HS, the transcription and translation of proteins involved in normal metabolic activities are reduced, and the synthesis of HSP proteins is stimulated; in addition, osmotic adjustment substances, such as inorganic ions, soluble sugars, proline, and betaine, accumulate to decrease heat-induced injury [48]. In this study, the response of red pitaya to HS was explored.

### 4.1. Roles of Heat-Responsive TFs

Previous studies have shown that HS signals are transduced through multiple signaling pathways to activate TFs, which induces the expression of many *HSPs* and other HS-responsive genes to respond to HS. TFs have an important effect on the response of plants to HS by regulating the expression of target genes. Studies have showed that there are more than 50 TFs families in plants, of which AP2/EREBP, NAC, MYB, WRKY, bHLH, HSF, and bZIP, are mainly involved in the response of plant to abiotic stress. AP2/EREBP is a large family of TFs found in plants, among which DREBs belong to the EREBP subfamily and participate in the response of plants to different abiotic stresses. For example, rice *OsDREB2B* and maize *ZmDREB2A* are induced to express in response to high temperature stress [49,50]. WRKYs is a kind of plant-specific transcription factor. WRKY protein can positively regulate the heat tolerance of plants. For instance, *CaWRKY40* participates in plant response to high temperature stresses in pepper, and rice *OsWRKY11* participates in resistance to heat shock stress [51,52]. NACs is also one of the plant-specific and largest transcription factor family, and involved in multiple processes in response to abiotic stress. Among them, wheat *TaNAC2L* can improve heat tolerance by regulating the expression of stress response genes [53]. 

The major TFs identified from the DEGs involved in response to HS in this study included MYBs (8), AP2/ERFs (5), bZIPs (2), MBF1C (1), HSPs (15), and HSFs (3), indicating that these TFs play a key role in response to HS. MBF1C is a highly conserved transcriptional coactivator and a key regulator of heat resistance. The expression of *DREB2A* and *HSFBs* under HS was reduced in an *mbf1c* mutant [54]. MYB TFs occur in all eukaryotes. MYBs are known to be involved in plant development, metabolism, and stress responses. To date, *AtMYB68*, *LeAN2*, and *OsMYB55* have been proposed to play an essential role in heat tolerance. In *Arabidopsis*, compared with WT plants, the *Atmyb68* mutant was significantly inhibited in vegetative growth at high temperature [55]. The overexpression of *LeAN2* in tomato resulted in anthocyanin accumulation and enhanced tolerance to HS by maintaining low levels of reactive oxygen species and high non-enzymatic antioxidant activity [56]. Overexpression of *OsMYB55* improves the tolerance of rice plants to high temperature by increasing the expression of the downstream genes *OsGS1;2*, *GAT1,* and *GAD3*, which are involved in amino acid metabolism [57]. In addition, transgenic *Arabidopsis* plants overexpressing *TaMYB80* showed enhanced tolerance to heat and drought stress, which might be due to the increased levels of cellular ABA [58]. In *Arabidopsis*, MYB30 regulates the response to HS through ANNEXIN (ANN)-mediated cytosolic calcium signals [59]. MYB30 binds to the promoters of ANN1 and ANN4 and inhibits their expression. Hereafter, ANNs regulate the increase in heat-induced [Ca^2+^]cyt, triggering downstream responses to HS, and *contig25252*, which encodes MYB30, was also upregulated in our study. The expression of seven other MYB TFs was markedly inhibited by HS (Table 3 and Table S6), indicating that they may play negative regulatory roles in response to HS.

HSPs are known to play key roles in protecting the cell metabolic apparatus as well as the response of plants to HS [1]. In this study, HSPs were markedly induced by HS (Table 4 and Table S6). The overproduction of Hsp70 confers resistance to heat and other types of abiotic stress in *Arabidopsis* [60]. Mitochondrial Hsp70 may suppress programmed cell death of rice protoplasts by inhibiting the amplification of reactive oxygen species [61]. In this study, the mRNA levels of *contig13847*, which belongs to the *Hsp70* gene family, were increased after heat treatment. HSFs are the terminal components of signal transduction and mediate the expression of HSPs and other HS-induced transcripts [62,63]. In addition, two DEGs (*contig26416* and *contig26417*) belonging to the HSF family were upregulated in this study. *HsfA2* plays an important role in preventing oxidative damage and cell death in plant organelles and is vital regulators of plant stress responses [64]. Overexpression *AtHsfA2* not only improved heat resistance but also improved the resistance to oxidative stress and hypoxia caused by salt [65]. Overall, the findings of this study enhance our understanding of the roles of HSPs and HSFs in response to HS in pitaya.

### 4.2. Mechanism Underlying the Response of Red Pitaya to HS

Various physiological processes, such as photosynthesis, respiration, transpiration, membrane thermostability, and osmotic regulation, are all adversely affected by HS. We conducted a comprehensive analysis of the transcriptome and metabolome to elucidate the regulatory networks of red pitaya involved in response to HS. The differential expressions of the most common heat-responsive genes were related to metabolic pathways (106 genes), biosynthesis of secondary metabolites (61 genes), carbon metabolism (17 genes), and biosynthesis of amino acids (11 genes) according to the KEGG analysis; similar results were also obtained from our analysis of metabolome data (Table S10). Meanwhile, the top 20 differentially expressed genes (Table 2) are mainly involved in photosynthesis and enzyme activity regulation in plant chloroplasts (such as contig32725, FER2; contig21847, PFE; and contig785, PFK3), and protein synthesis and activation of transporters (contig20699, contig2725, contig29974, etc.), which indicated that photosynthetic-related genes are more sensitive in response to heat stress.

Homeostasis, including the biosynthesis and compartmentalization of metabolites, is disturbed in plant tissues subjected to high temperatures [7]. Because heat tolerance is a polygenic trait, many biochemical and metabolic pathways are involved in the development and maintenance of heat tolerance, including antioxidant activity, membrane lipid unsaturation, gene expression and translation, protein stability, and the accumulation of compatible solutes [66]. In addition, flavonoids, anthocyanins, and plant steroids and other secondary metabolites play a considerable role in the response of plants to HS [1]. For example, HS in tomato plants causes the accumulation of soluble phenols; increases phenylalanine ammonia-lyase activity; and decreases the activity of peroxidase and polyphenol oxidase, which may be the mechanism of tomato plants to adapt to HS [67]. High temperature alters the activities of carbon metabolism enzymes, starch accumulation, and sucrose synthesis by downregulating specific genes involved in carbohydrate metabolism [10]. Moreover, “photosynthesis,” “MAPK signaling pathway,” and “plant hormone signal transduction” were also enriched (Figure 5, Table S8) according to the transcriptome analysis. Several lines of evidence suggest that plant growth regulators such as ABA, SA, ET, and BRs play a vital part in plant heat tolerance [62]. In tomato and Arabidopsis, brassinosteroids cause tolerance to heat stress by promoting the biosynthesis of major HSPs [68,69]. In sum, high temperatures have a negative impact on the photosynthesis, primary and secondary metabolism, and hormonal signal transduction and other physiological processes of red pitaya.

### 4.3. HuPR-1 Plays an Active Role in the Response to HS

The pathogenesis-related protein 1 (PR-1) gene family plays a significant part in response to biotic and abiotic stress in plants. To protect themselves against pathogens, plants have developed sophisticated mechanisms to adapt to their environment. Pathogenesis-related (PR) genes play essential roles in these mechanisms and are activated in response to pathogen attacks [70]. With their antifungal activities, PR-1 proteins are the main group of PR proteins induced by pathogens or salicylic acid. *PR-1* genes also play vital roles in response to abiotic stress. In wheat, *TaPR-1-1* expression is induced by freezing, salinity, and osmotic stress, and *TaPR-1-1* overexpression confers tolerance to these different types of stress in yeast and *Arabidopsis* [71]. *Di19* (drought-induced) upregulates the expression of pathogenesis-related *PR-1, PR-2,* and *PR-5* genes in *Arabidopsis* [72]. Thirteen novel *SlPR-1* genes were identified, each of which produce a protein belonging to the CAP superfamily in tomato, and drought stress leads to the upregulation of all *SlPR-1* genes (as high as 50-fold) [70]. In this study, the expression of *HuPR-1* was up-regulated up to 3.47-fold by HS (Table S6); the expression of this gene can also be induced by salt stress [37]. Overexpression of this gene greatly increased the tolerance of *Arabidopsis* to HS (Figure 8B,C), indicating that *HuPR-1* plays an active role in response to heat and salt stress. In short, the findings of this study provide new insights into the regulatory mechanism of heat stress that could aid future studies to examine the role of *PR-1* genes in response of plants to different types of abiotic stress.

## 5. Conclusions

We performed the transcriptomic and metabolomic analysis to characterize the molecular mechanism underlying the response of red pitaya to HS. The changes in numerous genes and metabolites indicated that the mechanisms involved in response to HS are complex and closely related in pitaya. 

## Figures and Tables

**Table 1:** De novo assembly and annotations metrics for the transcriptome of pitaya.

Item	Statistic Value
Total sequences	36,842
Total bases	78,871,914
Min sequence length	206
Max sequence length	40,937
Average sequence length	2141.82
Median sequence length	1749.00
N25 length	4210
N50 length	2848
N75 length	1799
N90 length	1107
N95 length	827
As	29.40%
Ts	29.33%
Gs	20.79%
Cs	20.48%
(A + T)s	58.72%
(G + C)s	41.28%
Ns	0.00%

**Table 2:** Top 20 DEGs in red pitaya after heat stress.

Gene ID	Log_2_ FC	*p*-Value	Annotation	
*Contig19129*	7.63	3.08 × 10^−6^	GRXC11; glutathione-disulfide oxidoreductase activity in the presence of NADPH and glutathione reductase.	up
*Contig20825*	7.26	2.29 × 10^−5^	NA	up
*Contig32725*	5.01	3.76 × 10^−10^	FER2, chloroplastic; stores iron in a soluble, non-toxic, readily available form. Important for iron homeostasis. Has ferroxidase activity.	up
*Contig32724*	4.98	1.03 × 10^−9^	N/A, chloroplastic; stores iron in a soluble, non-toxic, readily available form. Important for iron homeostasis. Has ferroxidase activity.	up
*Contig21847*	4.74	1.61 × 10^−8^	PFE, chloroplastic; stores iron in a soluble, non-toxic, readily available form. Important for iron homeostasis.	up
*Contig2380*	4.67	6.45 × 10^−14^	ELIP1, chloroplastic; early light-induced protein 1; prevents excess accumulation of free chlorophyll by inhibiting the entire chlorophyll biosynthesis pathway, and then prevents photooxidative stress; involved in seed germination	up
*Contig785*	4.63	1.25 × 10^−10^	PFK3; catalyzes the phosphorylation of D-fructose 6-phosphate to fructose 1,6-bisphosphate by ATP, the first committing step of glycolysis.	up
*Contig9300*	4.41	9.1 × 10^−4^	NA	up
*Contig3696*	4.39	1.63 × 10^−21^	PGM1, 2,3-bisphosphoglycerate-independent phosphoglycerate mutase; catalyzes the interconversion of 2-phosphoglycerate and 3-phosphoglycerate.	up
*Contig9996*	4.28	9.30 × 10^−6^	NA	up
*Contig9364*	−5.29	4.08 × 10^−5^	ART2; encoded on the antisense strand of the nuclear 25S rDNA.	down
*Contig17684*	−5.49	6.21 × 10^−5^	PER72, peroxidase 72; removal of H_2_O_2_; oxidation of toxic reductants; biosynthesis and degradation of lignin; suberization; auxin catabolism; response to environmental stresses such as wounding, pathogen attack, and oxidative stress.	down
*Contig7023*	−5.51	1.27 × 10^−8^	NA	down
*Contig2725*	−6.29	3.73 × 10^−18^	rbgA, ribosome biogenesis; GTPase A; essential protein that is required for a late step of 50S ribosomal subunit assembly.	down
*Contig16950*	−6.58	1.09 × 10^−7^	NA	down
*Contig18702*	−6.91	6.00 × 10^−4^	ALMT2, aluminum-activated malate transporter 2.	down
*Contig31875*	−7.22	1.92 × 10^−31^	LBD41, LOB domain-containing protein 41; cellular response to hypoxia; regulation of transcription.	down
*Contig29974*	−8.03	2.66 × 10^−5^	ALMT10, aluminum-activated malate transporter 10.	down
*Contig20699*	−8.98	1.78 × 10^−12^	RAB15, glycine-rich RNA-binding, abscisic acid-inducible protein.	down
*Contig352*	−10.55	4.34 × 10^−18^	NA	down

NA, no annotation.

**Table 3:** MYB proteins regulated by heat stress in red pitaya.

Gene ID	Log_2_ FC	Functional Description	Homology	Species
*Contig9674*	−1.66	MYB domain-containing protein	MYBB	*Xenopus laevis*
*Contig26304*	−1.58	MYB domain-containing protein	MYBB	*Xenopus laevis*
*Contig25078*	−2.38	MYB-related protein 306	MYB06	*Antirrhinum majus*
*Contig25077*	−2.70	MYB-related protein 306	MYB06	*Antirrhinum majus*
*Contig25252*	1.66	MYB domain protein 30	MYB30	*Antirrhinum majus*
*Contig28175*	−1.79	MYB domain protein 44	MYB44	*Arabidopsis thaliana*
*Contig28176*	−1.77	MYB domain protein 44	MYB44	*Arabidopsis thaliana*
*Contig21877*	−1.37	MYB domain protein 86	MYB86	*Arabidopsis thaliana*

**Table 4:** Heat-shock proteins and heat transcription factors regulated by heat stress in red pitaya.

Gene ID	Log_2_ FC	Functional Description	Homology	Species
*Contig26416*	1.86	Heat shock factor A2	HsfA2	*Arabidopsis thaliana*
*Contig26417*	2.54	Heat shock factor A2	HsfA2	*Arabidopsis thaliana*
*Contig27952*	1.67	Heat shock factor R2	HspR2	*Arabidopsis thaliana*
*Contig20486*	1.13	Heat shock protein 83	Hsp83	*D. melanogaster*
*Contig1864*	3.72	Heat shock protein 16	Hsp16	*Glycine max*
*Contig1898*	4.91	Heat-shock cognate 71 kDa protein	Hsp7C	*Petunia hybrida*
*Contig1899*	1.43	Heat-shock cognate 71 kDa protein	Hsp7C	*Petunia hybrida*
*Contig3506*	2.20	Heat-shock cognate 71 kDa protein	Hsp7C	*Petunia hybrida*
*Contig13687*	3.89	Heat shock protein 41	Hsp41	*Glycine max*
*Contig1021*	4.67	Heat shock protein 83	Hsp83	*Ipomoea nil*
*Contig1022*	2.34	Heat shock protein 83	Hsp83	*Ipomoea nil*
*Contig1023*	2.27	Heat shock protein 83	Hsp83	*Ipomoea nil*
*Contig27132*	6.29	Heat-shock cognate 71 kDa protein	Hsp7C	*Rattus norvegicus*
*Contig22611*	1.37	70 kDa heat shock-related protein	Hsp7S	*Pisum sativum*
*Contig20223*	−1.94	heat shock 70 kDa protein cognate 5	Hsp7E	*Spinacia oleracea*
*Contig26216*	−1.31	Heat shock protein 72	Hsp72	*Solanum lycopersicum*
*Contig14935*	4.25	22.0 kDa class IV heat shock protein	Hsp22	*Ipomoea nil*
*Contig13847*	1.59	Heat shock protein 70	Hsp70	*Arabidopsis thaliana*

**Table 5:** KEGG classification of significantly enriched pathways, following metabolite analysis of differentially accumulated metabolites.

ID ^a^	Term	Diff_Metabolites	Metabolite_id
ko00970	Aminoacyl-tRNA biosynthesis	2	meta_51; meta_10
ko00630	Glyoxylate and dicarboxylate metabolism	2	meta_40; meta_8
ko00960	Tropane, piperidine, and pyridine alkaloid biosynthesis	1	meta_10
ko00460	Cyanoamino acid metabolism	1	meta_10
ko00020	Citrate cycle (TCA cycle)	1	meta_40
ko00240	Pyrimidine metabolism	1	meta_212
ko00966	Glucosinolate biosynthesis	1	meta_10
ko01200	Carbon metabolism	2	meta_51; meta_8
ko00660	C5-Branched dibasic acid metabolism	2	meta_8; meta_40
ko01110	Biosynthesis of secondary metabolites	2	meta_10; meta_40
ko00260	Glycine, serine, and threonine metabolism	1	meta_51
ko02010	ABC transporters	1	meta_10
ko00280	Valine, leucine, and isoleucine degradation	1	meta_10
ko01210	2-Oxocarboxylic acid metabolism	3	meta_8; meta_10; meta_40
ko00290	Valine, leucine, and isoleucine biosynthesis	2	meta_10; meta_8
ko01230	Biosynthesis of amino acids	2	meta_51; meta_10
ko00270	Cysteine and methionine metabolism	1	meta_51
ko01100	Metabolic pathways	5	meta_212; meta_8; meta_51; meta_40; meta_10

^a^ Pathway-map ID. In the KEGG database (http://www.genome.jp/kegg/, accessed on 10 March 2021). Term, annotation of various metabolic pathways in plants; Diff_Metabolites, the number of differentially expressed compounds under heat treatment conditions compared with the control group.

## Data Availability

The deep sequencing data of total RNA were submitted to NCBI Sequence Read Archive (SRA) with accession number Bioproject: PRJNA747865.

## Supplementary Materials

The following are available online at https://www.mdpi.com/article/10.3390/genes12111714/s1, Figure S1: RNA-Seq dataset validation by using qRT-PCR. Seven DEG contigs (*x*-axis) were randomly selected from the RNA-Seq dataset and validated by qRT-PCR. The *y*-axis displays natural log_2_-transformed fold changes for both datasets. *HuUBQ* was used as the internal standard. Mean values and SDs of three biological replicates are shown. Figure S2: GO classifications of DEGs using the PANTHER database. The obtained 1434 annotated DEGs were divided into three GO categories: molecular function (A), biological process (B), and cellular component (C). The percentages shown are based on the numbers of genes in a given GO category. Table S1: Primers used in this study. Table S2: Dragon_fruit_rnaseq_count_heat_stress at 0, 24, and 72 h. Table S3: Dragon_fruit_trinity_combined_v2_fasta_cap_contigs_uniprot_annotation. Table S4: Dragon_fruit_trinity_combined_v2_fasta_cap_contigs_blastn. Table S5: CDS amplification and sequencing results for confirm the sequence assembly. Table S6: DEGs genes annotation. Table S7: All DEG pathways in KEGG KAAS-2. Table S8: All DEG pathways in KEGG-2. Table S9: N0_vs_N24 metabolites in this study. Table S10: Integrated analysis between differentially accumulated metabolites and DEGs.
