# Supplementary material for: An Integrative Transcriptomic and Metabolomic Analysis of Red Pitaya (Hylocereus polyrhizus) Seedlings in Response to Heat Stress
Source: Genes (Basel). 2021 Oct 27;12(11):1714. doi: 10.3390/genes12111714 (PMC8625689; doi:10.3390/genes12111714)
Supplement: Supplementary file 1 [file genes-12-01714-s001.zip › Table S10 Primers used in this study.pdf]

Supplementary Table S10

Primer ID	Sequence (from 5' to 3')	Purpose
HuPR-1-F1	ATGAAGCCCTATAATTTCTTCCT	Amplify CDS of *HuPR-1*
HuPR-1-R1	ATACGGACGTTCACCTATAACATTG	
HuPR-1-F2	TGACCATGGTAGATCTGATGAAGCCCTATAATTTCTTCCT	Amplify CDS for *HuPR-1*-OE by homologous recombination construction, *Bgl*II and *Spe*I sites were underlined
HuPR-1-R2	CTTCTCCTTTACTAGTATACGGACGTTCACCTATAACAT	
HuPR-1-RT-F	AATGCCTCCTATGCTAAAATCTACG	gene primer pair for qRT-PCR of *HuPR-1*
HuPR-1-RT-R	CGAGCCACCTCCCCAAAAT	
1302-F	ACTCAATGACAAGAAGAAAATCT	gene primer pair for genomic PCR to confirm *HuPR-1*-OE transgenic plants.
1302-R	TCACCTTCACCCTCTCCACT	
HuUBQ-RT-F	CAATGCTGGGCTCCACTGC	gene primer pair for qRT-PCR of reference gene *HuUBQ* in *Pitaya*
HuUBQ-RT-R	AATAATGGGCCCTGCAGATAGC	
9489-RT-F	AGGATTGTGGAAGCAGGTTTT	gene primer pair for qRT-PCR of *Contig9489*.
9489-RT-R	ACTCGTGCCCGTTGATTGA	
34259-RT-F	GACTTCTATGTCAACGCCACCT	gene primer pair for qRT-PCR of *Contig34259*.
34259-RT-R	TGATACTACTGCCACTGCTACCC	
16948-RT-F	ACAGGGTGTTGCTTCATCATTC	gene primer pair for qRT-PCR of *Contig16948*.
16948-RT-R	GCCTATTCGGATTCTCAGTTTG	
33961-RT-F	AACCTGCCAACCAACCTATCA	gene primer pair for qRT-PCR of *Contig33961*.
33961-RT-R	TTTCGTGGGACCATTACCG	
28068- RT-F	GGCACCCAAATCAATACG	gene primer pair for qRT-PCR of *Contig28068.*
28068- RT-R	GGAGCACAACAACAAAGC	
28613-RT-F	GTGGGGAAGGGAATAAGGAGC	gene primer pair for qRT-PCR of *Contig28613*.
28613-RT-R	GGAGGAAACTGAAACACTGTGACTA	
35270-RT-F	ATTGGAGGCGATGGGACAC	gene primer pair for qRT-PCR of *Contig35270*.
35270-RT-R	TTGCACGTTGGGCTTCTTC	
