# Supplementary material for: An Integrative Transcriptomic and Metabolomic Analysis of Red Pitaya (Hylocereus polyrhizus) Seedlings in Response to Heat Stress
Source: Genes (Basel). 2021 Oct 27;12(11):1714. doi: 10.3390/genes12111714 (PMC8625689; doi:10.3390/genes12111714)
Supplement: Supplementary file 1 [file genes-12-01714-s001.zip › Table S4 CDS amplification and sequencing results for confirm the sequence assembly.pdf]

Supplementary Table S4. CDS amplification and sequencing results for confirm the sequence assembly

Gene ID	CDS length in RNA-Seq (bp)	Sequencing length (bp)	Sequencing cover (%)	Identity (%)
Contig34259	1029	912	89	100
Contig34203	1038	894	86	100
Contig10820	594	594	100	100
Contig23583	1572	1572	100	100
Contig7784	1464	1464	100	99
Contig39932	1126	1126	91	100
Contig23977	1257	1257	100	100
Contig34259	888	888	100	100
Contig9489	1554	1554	100	100
