# Supplementary figures and images for: An Integrative Transcriptomic and Metabolomic Analysis of Red Pitaya (Hylocereus polyrhizus) Seedlings in Response to Heat Stress
Source: Genes (Basel). 2021 Oct 27;12(11):1714. doi: 10.3390/genes12111714 (PMC8625689; doi:10.3390/genes12111714)

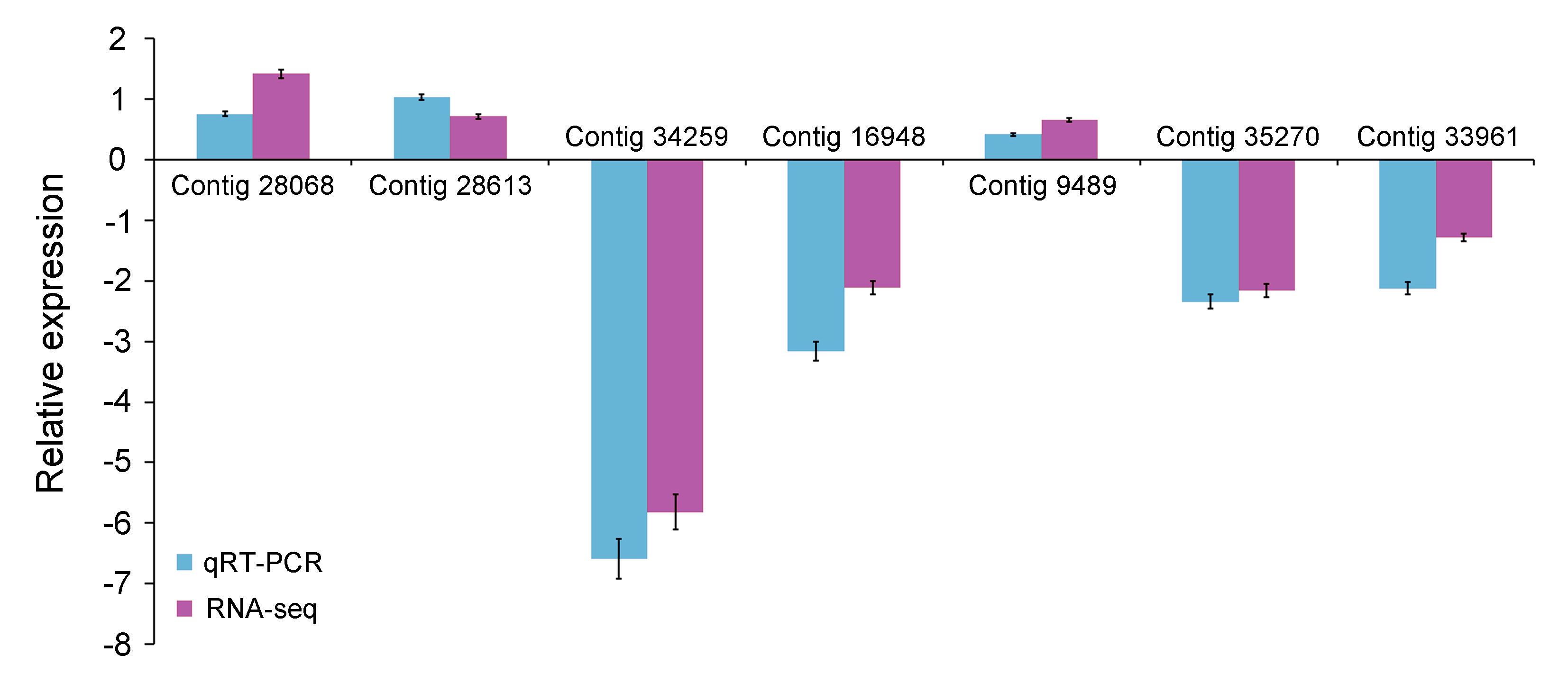

Supplement: Supplementary file 1 [file genes-12-01714-s001.zip › FigureS1 RNA-Seq dataset validation by using qRT-PCR.tif]

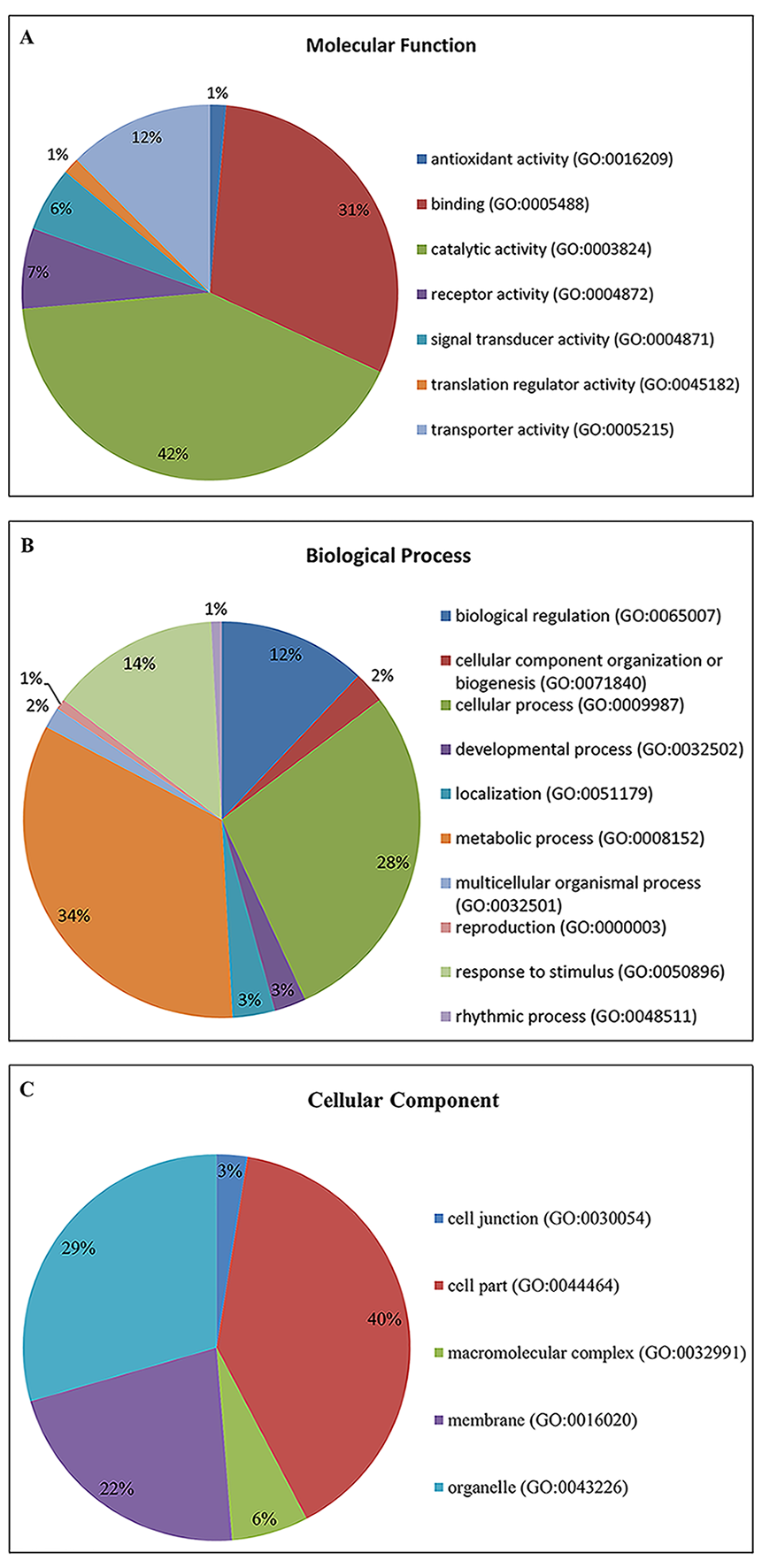

Supplement: Supplementary file 1 [file genes-12-01714-s001.zip › FigureS2 GO classifications of DEGs using the PANTHER database.tif]
